# Shifting from Immunohistochemistry to Screen for *ALK* Rearrangements: Real-World Experience in a Large Single-Center Cohort of Patients with Non-Small-Cell Lung Cancer

**DOI:** 10.3390/cancers16122219

**Published:** 2024-06-14

**Authors:** Marius Ilié, Samantha Goffinet, Guylène Rignol, Virginie Lespinet-Fabre, Salomé Lalvée, Olivier Bordone, Katia Zahaf, Christelle Bonnetaud, Kevin Washetine, Sandra Lassalle, Elodie Long-Mira, Simon Heeke, Véronique Hofman, Paul Hofman

**Affiliations:** 1 Laboratory of Clinical and Experimental Pathology, Pasteur Hospital, Nice University Hospital, FHU OncoAge, IHU RespirERA, 06000 Nice, France; ilie.m@chu-nice.fr (M.I.); goffinet.s@chu-nice.fr (S.G.); rignol.g@chu-nice.fr (G.R.); lespinet-fabre.v@chu-nice.fr (V.L.-F.); lalvee.s@chu-nice.fr (S.L.); zahaf.k@chu-nice.fr (K.Z.); bonnetaud.c@chu-nice.fr (C.B.); washetine.k@chu-nice.fr (K.W.); lassalle.s@chu-nice.fr (S.L.); long-mira.e@chu-nice.fr (E.L.-M.); hofman.v@chu-nice.fr (V.H.); 2 Hospital-Integrated Biobank (BB-0033-00025), Pasteur Hospital, Nice University Hospital, FHU OncoAge, IHU RespirERA, 06000 Nice, France; bordone.o@chu-nice.fr; 3 Department of Thoracic/Head & Neck Medical Oncology, The University of Texas MD Anderson Cancer Center, Houston, TX 77030, USA; sheeke@mdanderson.org

**Keywords:** *ALK* rearrangement, immunohistochemistry, fluorescent *in situ* hybridization, next generation sequencing, shifting

## Abstract

**Simple Summary:**

Identifying *ALK* fusions in advanced non-small-cell lung carcinoma (aNSCLC) is mandatory for targeted therapy. Current methods use ALK IHC, *ALK* FISH, or NGS, but face challenges due to low fusion frequency, possible tissue exhaustion, and test limitations. We compared RNA NGS with ALK IHC and *ALK* FISH in 1246 NSCLC cases, finding RNA NGS faster and equally effective. This suggests the need to replace systematic ALK IHC with RNA NGS reflex testing for more efficient assessments of *ALK* status.

**Abstract:**

The identification of *ALK* fusions in advanced non-small-cell lung carcinoma (aNSCLC) is mandatory for targeted therapy. The current diagnostic approach employs an algorithm using ALK immunohistochemistry (IHC) screening, followed by confirmation through *ALK* FISH and/or next-generation sequencing (NGS). Challenges arise due to the infrequency of *ALK* fusions (3–7% of aNSCLC), the suboptimal specificity of ALK IHC and *ALK* FISH, and the growing molecular demands placed on small tissue samples, leading to interpretative, tissue availability, and time-related issues. This study investigates the effectiveness of RNA NGS as a reflex test for identifying *ALK* fusions in NSCLC, with the goal of replacing ALK IHC in the systematic screening process. The evaluation included 1246 NSCLC cases using paired techniques: ALK IHC, *ALK* FISH, and *ALK* NGS. ALK IHC identified 51 positive cases (4%), while RNA NGS detected *ALK* alterations in 59 cases (4.8%). Of the 59 *ALK*-positive cases identified via NGS, 53 (89.8%) were confirmed to be positive. This included 51 cases detected via both FISH and IHC, and 2 cases detected only via FISH, as they were completely negative according to IHC. The combined reporting time for ALK IHC and *ALK* FISH averaged 13 days, whereas ALK IHC and RNA NGS reports were obtained in an average of 4 days. These results emphasize the advantage of replacing systematic ALK IHC screening with RNA NGS reflex testing for a more comprehensive and accurate assessment of *ALK* status.

## 1. Introduction

Detecting *ALK* rearrangements in stage IIIB/IV non-squamous non-small-cell lung cancer (NS-NSCLC) patients is pivotal for their eligibility for targeted therapy, particularly involving first- and second-generation ALK tyrosine kinase inhibitors [1,2].

Hence, there is a pressing need to systematically screen for *ALK* rearrangements in cases of advanced non-squamous NSCLC (aNS-NSCLC) at baseline [3].

For years, FISH analysis has been the benchmark method for detecting *ALK* rearrangements, while it is a time-consuming process requiring specialized equipment and high expertise for interpreting various signal variants. *ALK:EML4* fusions often exhibit a short break pattern, leading to potential misinterpretation and false-negative results, though this can be mitigated by using the *ALK::EML4* fusion probe [4].

Some samples fail to meet FISH analysis criteria due to various quality-related issues and a very low percentage of tumor cells. With the advent of high-performance rabbit monoclonal antibodies, immunohistochemistry (IHC) has become a valuable alternative to FISH. While easier to interpret and not requiring specialized equipment like FISH, IHC cannot replace FISH unless standardized and properly validated [2,5].

Despite the high concordance rate between IHC and FISH, routine practice sometimes yields discordant results due to technical issues and the diverse properties of ALK protein produced by different *ALK* fusion variants [6,7].

Moreover, next-generation sequencing (NGS) can detect *ALK* fusion in FISH-negative cases, with NGS recently overtaking FISH as the predominant *ALK* testing method [8,9]. This trend is anticipated to persist, given that multiple NGS methodologies can identify a wide range of actionable gene alterations. Current guidelines from ESMO and NCCN for NSCLC advocate for extensive molecular profiling, reinforcing this practice [3,10]. The continuous discovery of therapeutic molecular targets for NS-NSCLC has increased the number of predictive biomarkers that need to be evaluated before any treatment. These biomarkers include *EGFR*, *ALK*, *ROS1*, *BRAF*, *RET*, *NTRK*, *MET*, *KRAS*, and *HER2* [3]. However, in routine clinical practice, sequentially testing for genomic alterations in each of these genes poses several challenges. Firstly, the turnaround times (TATs) needed to obtain the results can significantly delay the initiation of targeted treatments. Secondly, some tests may be impractical or yield uncertain or false-negative results, particularly when dealing with small tissue biopsies or samples with low tumor content. This can lead to insufficient amounts of extracted nucleic acid or an inadequate number of visible tumor cells on tissue sections [11].

Currently, the utility of IHC for ALK screening is under scrutiny, paving the way for RNA NGS reflex testing, capable of examining all necessary genomic alterations associated with available targeted therapies in a single step in routine clinical practice [3].

This study aimed to compare *ALK* rearrangement screening results using IHC and RNA NGS in a large single-center cohort of NS-NSCLC patients, with *ALK* FISH results as a reference. Additionally, we compared the TATs for obtaining these results.

## 2. Patients and Methods

**Figure 1:**
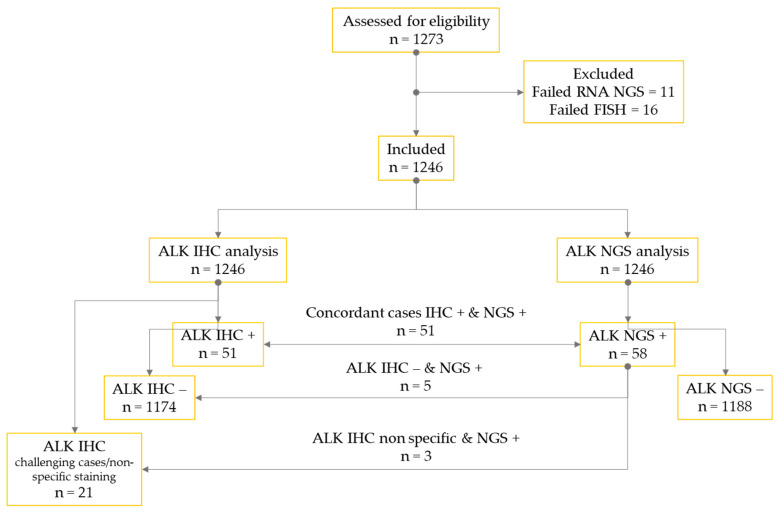
CONSORT flow diagram of the study.

Between January 2016 and December 2023, 1246 NS-NSCLC consecutive patients were tested systematically via ALK IHC, and DNA and RNA NGS (Laboratory of Clinical and Experimental Pathology, IHU RespirERA, Nice, France; Figure 1).

Diagnoses were made on a morphological and IHC basis according to the recommendations of the 2015 WHO classification for lung cancer, with the IASLC/ATS/ERS-recommended modifications, by senior lung pathologists (M.I., E.L, S.L, V.H., and P.H.) [12].

The TAT was defined as the duration between receiving the final histological diagnosis reports and the electronic validation of the molecular reports.

All tumor specimens were used with informed signed consent from the patients. The study was approved by the local ethics committee (Human Research Ethics Committee, Nice University Hospital Center/Hospital-related Biobank BB-0033-00025; http://www.biobank-cotedazur.fr/, accessed on 15 March 2024) and was performed in accordance with the guidelines of the Declaration of Helsinki.

### 2.1. ALK Immunohistochemistry

FFPE sections were freshly cut, resulting in a thickness of 4 μm. Within a maximum of one day after sectioning, the slides were stained using IHC. All IHC staining procedures were carried out on a Ventana BenchMark Ultra automated immunostainer (Ventana Medical Systems, Roche Group, Tucson, AZ, USA) [5,13,14]. Following CC1 antigen retrieval, tissue sections were incubated with the primary rabbit monoclonal antibody anti-ALK (Clone D5F3, prediluted, Ventana Medical Systems). OptiView DAB IHC Detection Kit (Ventana Medical Systems) and OptiView Amplification Kit (Ventana Medical Systems) were used according to the manufacturer’s technical instructions to visualize the bound anti-ALK primary antibody. ALK-Lung Analyte Control (HCL009, HistoCyte Laboratories, Newcastle upon Tyne, UK) was used as a positive external control for all tests.

All the procedures were accredited in the laboratory under the ISO 15189 norm [15] (COFRAC n°8-3034).

Every specimen was examined and evaluated by five pathologists (M.I., E.L.M., S.L., V.H., and P.H.). The Ventana ALK Scoring Interpretation Guide was used for the interpretation of the ALK IHC assay. Cases were scored positive if strong granular cytoplasmic brown staining in tumor cells (any percentage of positive tumor cells) was present [5]. After initial calibration according to the interpretation guide and conducting annual internal quality controls for ALK IHC interpretation, the concordance rate among the five pathologists reached 100% in this study.

### 2.2. ALK Fluorescence In Situ Hybridization

The Abbott ALK break-apart probe (Vysis LSI ALK Dual Color; Abbott Molecular, Rungis, France) was used on 4 µm thick FFPE tissue sections, according to the manufacturer’s technical instructions, as detailed in previous studies [16]. Every specimen was examined and evaluated by one senior pathologist (E.L.M.). This ALK FISH assay was accredited in the laboratory according to the ISO 15189 norm (COFRAC n°8-3034).

### 2.3. Ultra-Fast Next-Generation Sequencing

Patients included in the study underwent fast DNA- and RNA-based NGS as a form of reflex testing at the LPCE (Nice University Hospital, France), accredited under the ISO 15189 vs. 2022 norm for somatic genomic testing by NGS in routine clinical practice (www.cofrac.fr, accessed on 15 March 2024).

Nucleic acids were extracted using Maxwell RSC Instrument (Promega, Madison, WI, USA) with the Maxwell RSC FFPE Plus DNA kit or the Maxwell RSC RNA FFPE kit (Promega). Concentrations were measured using the Qubit Fluorometric quantification assay (Thermo Fisher Scientific, Waltham, MA, USA) with Qubit RNA HS Assay Kit and Qubit dsDNA HS Assay Kit.

Genomic alterations were detected using ion semiconductor sequencing (Ion Torrent™ Technology, Thermo Fisher Scientific) on Ion Torrent™ Genexus™ Integrated Sequencer, following the manufacturer’s technical instructions, as previously described [17,18]. The Oncomine™ Precision Assay GX (OPA, Thermo Fisher Scientific, catalog number A46291) was used, covering 50 key genes—45 for DNA mutations, 18 for fusions, and 14 for copy number variants (CNVs), including a 5′/3′ expression imbalance caller for novel fusion detection. The Genexus sequencer could sequence up to 16 samples per run. [17,18].

## 3. Results

The main epidemiological, clinical, and pathological data of the enrolled patients are shown in Table 1.

**Figure 2:**
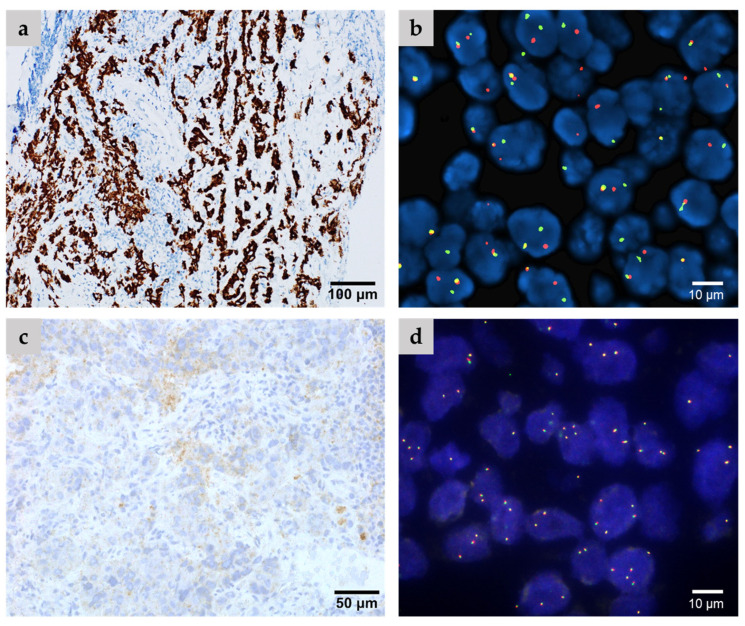
Representative cases include (**a**,**b**) an ALK-positive case confirmed via both IHC (**a**) and FISH analysis (**b**), as well as (**c**,**d**) an ALK-negative case demonstrating non-specific staining according to IHC (**c**) and negative FISH analysis (**d**).

ALK IHC was assessed in 1246 cases of NS-NSCLC, revealing unequivocal positive staining in 51 out of 1246 cases (4%) (Figure 2).

*ALK* FISH analysis yielded positive results in 53 out of 1246 cases (4.2%). Among these cases, there were two cases where non-specific staining was observed and recorded as negative by IHC, but *ALK* 5′/3′-end expression imbalance was detected via RNA NGS.

**Figure 3:**
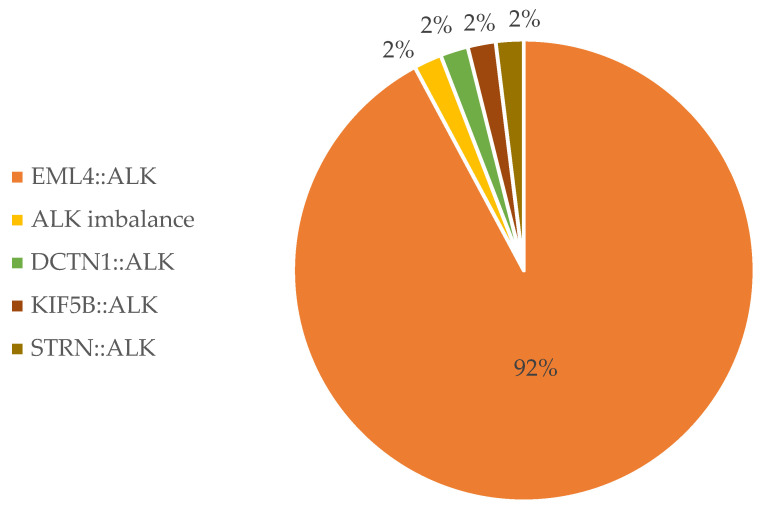
RNA NGS-detected *ALK* fusions in the study.

RNA NGS analysis identified *ALK* alterations in 59 out of 1246 cases (4.7%) (Figure 3).

**Figure 4:**
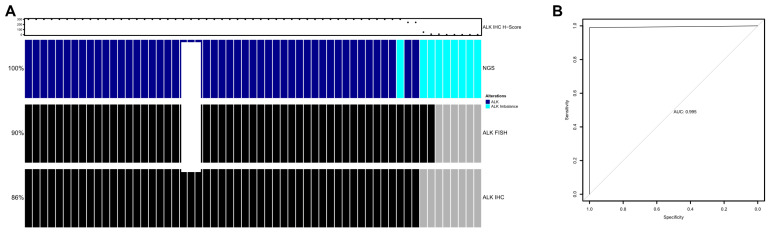
(**A**) Overview of *ALK* fusion detection using NGS (*ALK*-positive, dark blue; *ALK* 5′/3′-end expression imbalance, turquoise), *ALK* FISH (*ALK*-positive, black; ALK-negative, gray), and ALK IHC (ALK-positive, black; ALK-negative, gray). Each column represents one patient. The IHC H-score is shown above. (**B**) Receiver operator characteristic (ROC) curve for the detection of *ALK* via NGS compared with IHC H-score in the full cohort of 1246 patients.

A positive *ALK* result of NGS analysis was confirmed in 53 out of 59 cases (89.8%), comprising 51 cases identified via both FISH and IHC, and 2 cases detected solely via FISH due to completely negative IHC results (Figure 4A). Additionally, five cases were false positives according to RNA NGS, showing *ALK* 5′/3′-end expression imbalance, but tested negative via both FISH and IHC, while one case was FISH-negative but exhibited non-specific staining under IHC (Figure 4A).

In the full cohort, the area under the curve using the receiver operating characteristic (ROC) curve was 0.995. Based on the Youden J method, an optimal cut-off was defined as an H-score of 145. At this cutoff, specificity was 98.99%, sensitivity was 100%, NPV was 100%, and PPV was 98.04% for the *ALK* RNA NGS test compared with IHC (Figure 4B).

The average TAT to obtain DNA and RNA NGS reports was 4 days, ranging from 3 to 8 days. For both the ALK IHC and *ALK* FISH reports, the total mean TAT was 13 days. Specifically, the ALK IHC report took an average of 2 days (ranging from 2 to 5 days), while the *ALK* FISH report took an average of 13 days (ranging from 6 to 21 days).

## 4. Discussion

This study demonstrated that implementing an ultrafast DNA and RNA NGS approach at baseline can significantly enhance reflex testing for *ALK* rearrangements in NS-NSCLC.

Overall, our findings prompted us to discontinue the ALK IHC screening approach and the sequential ALK IHC/*ALK* FISH algorithm in our laboratory in favor of the RNA NGS approach. We also emphasize the importance of FISH analysis as the preferred orthogonal method for confirming ambiguous *ALK* results showing *ALK* imbalance identified via RNA NGS.

Among the patients with NSCLC, *ALK* gene alterations occur in approximately 3–7% of cases, consistent with findings from previous series [19,20]. Our study cohort demonstrated a similar prevalence of *ALK* alterations as reported in NSCLC patients [19,20]. DNA and RNA NGS results were obtained within an average of four working days. In contrast, ALK IHC results were available within an average of 2 working days, but confirmation of positivity required an additional 13 working days (a range of 6–21 days) under *ALK* FISH.

The decision to move away from routine ALK IHC screening in patients with NS-NSCLC was justified by various factors, particularly based on the findings of our study. ALK status assessment through IHC has limitations, as evidenced by several false-negative results that were confirmed by discrepancies with *ALK* FISH results [21]. Furthermore, the choice of anti-ALK antibody is crucial due to the variability in specificity and sensitivity among commercial antibodies. The ALK (D5F3) CDx IHC assay stands out as the sole IHC assay approved by the US Food and Drug Administration for the standalone testing of *ALK* rearrangements in NSCLC, owing to its high performance and clinical validation [22]. Finally, in numerous cases, both ALK IHC and *ALK* FISH are redundantly performed, leading to increased associated costs, increased workload in the pathology laboratory, and longer TATs for obtaining reports. Importantly, this practice can also deplete tumor tissue reserves, especially in cases of small biopsies or when tumor cell percentages are low [23].

Our findings showed excellent agreement between *ALK* FISH results and those obtained from RNA NGS. RNA NGS was conducted concurrently with DNA NGS testing, enabling a comprehensive assessment of essential gene statuses at the baseline for NS-aNSCLC.

NGS offers the ability to analyze a broad panel of driver alterations simultaneously, requiring a smaller specimen volume compared with sequential analysis of driver oncogenes like *EGFR*, *KRAS*, *BRAF*, *HER2*, *MET*, *ALK*, *ROS1*, *NTRK*, and *RET* [24]. There are two primary NGS approaches: DNA-based NGS and hybrid capture-based NGS. DNA-based NGS can target predefined breakpoints. The reported sensitivity and specificity of DNA-based NGS assays for detecting *ALK* rearrangements in NSCLC diagnosed via FISH and IHC are 85% and 79%, respectively [25]. In contrast, hybrid capture-based NGS has the capability to analyze most breakpoints, including those that are unknown [26].

Previously, a significant limitation of relying solely on NGS for evaluating ALK rearrangements was the delay in obtaining results. This delay could hinder the timely administration of targeted therapy due to issues related to sample organization, workflow, and sequencing methods [24]. However, the development of a new ultra-fast NGS system has revolutionized this process, delivering results within an average of four days for positive cases. This surpasses the turnaround time of sequentially using ALK IHC followed by ALK FISH [17,18,27]. It is important to note that RNA NGS can identify all fusion partners of the *ALK* gene and can enable the detection of short deletions that might not be detectable with *ALK* FISH [28,29,30]. Although DNA-based sequencing can identify rearrangements of fusion genes within intron regions, these sequences often differ from messenger RNA (mRNA) fusions. This underscores the fact that RNA NGS is indeed the preferred approach for evaluating the ALK status [31]. Furthermore, it is important to highlight that in certain cases, *ALK* fusions are detected through imbalance assessment, which can be confusing due to its lower specificity [21]. If an *ALK* fusion is identified through expression imbalance or with a low number of reads, it is recommended to conduct orthogonal testing using FISH to enhance specificity [27,32].

Interestingly, capture-based NGS can detect *ALK* rearrangements in blood samples noninvasively with high sensitivity and specificity. This approach also enables the monitoring of tumor dynamics and identification of drug-resistant mutations [33].

There are several approaches to target enrichment for RNA next-generation sequencing (NGS), including amplicon-based, hybrid capture, and anchored multiplex methods. Each has its advantages and disadvantages [34].

Amplicon-based enrichment has high sensitivity for detecting known variants, is quick and cost-effective, and requires relatively small amounts of input RNA. However, it is limited to detecting known variants and is less effective for novel or complex rearrangements. It also has the potential for amplification bias and a limited dynamic range [34].

Hybrid capture can detect a broad range of variant types, including novel fusions and splice variants. It offers high specificity and coverage uniformity, making it suitable for high-throughput applications. On the downside, it is more expensive and time-consuming compared with amplicon-based methods, requires larger amounts of input RNA, and can potentially capture off-target sequences, leading to noise [34].

Anchored multiplex PCR is effective for identifying gene fusions, including those with unknown partners, and has a flexible design that allows for the detection of various types of rearrangements. It offers high sensitivity and specificity. However, it involves complex design and optimization, is costlier compared with amplicon-based methods, and requires a moderate amount of input RNA [34].

The Oncomine assay, an example of an anchored multiplex PCR method, is specifically designed for detecting gene fusions and variants in cancer research. It has high sensitivity and specificity for detecting a wide range of gene fusions, including those with unknown partners [35]. It features a streamlined workflow and comprehensive coverage of relevant cancer genes, and is suitable for use with limited RNA samples, such as those obtained from formalin-fixed, paraffin-embedded (FFPE) tissues. However, the assay can be relatively expensive compared with simpler methods and requires careful design and optimization to ensure comprehensive coverage and accurate detection. While effective for fusion detection, it may be less suitable for detecting other types of variants or for applications requiring whole-transcriptome analysis. Additionally, interpreting the results can be complex and may require specialized bioinformatics tools and expertise [35].

Simultaneous performance of DNA and RNA NGS enables comprehensive assessment of various genomic alterations that are essential for baseline evaluation in NS-NSCLC. This approach facilitates the investigation of key genetic changes necessary for understanding the molecular profile of aNS-NSCLC at the outset [3].

In this context, it is important to highlight that the simultaneous presence of driver oncogene mutations along with an *ALK* rearrangement may have implications for predicting diverse clinical outcomes in patients with aNS-NSCLC [36]. Nevertheless, several constraints may hinder the implementation of the new algorithm described above in routine clinical practice. First, unlike IHC platforms, which are widely available in most pathology laboratories, NGS approaches are not uniformly accessible across all countries or even within organizations and institutions within a single country [37]. Second, even if NGS approaches are accessible, their use may be limited due to the high costs and possible lack of reimbursement, which can result in longer TATs for obtaining results that may not align with international guidelines [38]. Considering their reduced expenses and shorter TATs, IHC and/or rapid RT-PCR can be viable alternatives for evaluating *ALK* status [37,39,40]. Notably, in small biopsies or when there is an extremely low content of tumor cells, the IHC and FISH methods can be indispensable as they might be the sole methods capable of detecting therapeutic targets. This is particularly worrisome as NGS might produce false-negative results in cases of inadequate nucleic acid quantity and/or quantity [41]. Unlike RNA NGS, the *ALK* FISH assay might yield false-negative results, especially in cases with small deletions [42]. Additionally, *ALK* FISH entails relatively higher costs, and technical challenges, requiring a sufficient number of tumor cells, and can be labor-intensive.

Therefore, the integration of diverse approaches for assessing *ALK* status within pathology laboratories is crucial. *ALK* FISH serves as a valuable orthogonal tool alongside ALK IHC to confirm doubtful NGS and/or RT-PCR results, particularly in young and/or non-smoking patients. Furthermore, cutting-edge *in situ* technologies like multiplex IHC, incorporating various antibodies including anti-ALK antibodies, can conserve tissue material and expedite TATs by simultaneously providing results for different targetable molecules.

## 5. Conclusions

In conclusion, our study underscores the recommendation to discontinue IHC for evaluating *ALK* rearrangement status in NS-NSCLC in favor of adopting ultrafast RNA NGS reflex testing as the preferred method. However, *ALK* FISH remains valuable for validating diagnoses in cases of uncertain NGS results or when dealing with very small tissue biopsies containing few tumor cells. Additionally, *ALK* FISH may be considered in specific circumstances, such as with young and/or non-smoker patients, or based on specific requests from physicians.

## Figures and Tables

**Table 1:** Main epidemiological and pathological data of the 1246 patients with NS-NSCLC included in the study.

Variable	Type	n (%)
All patients (n = 1246)		
Age [median (range)]	68 (25–96)	
Gender		
	Male	716 (57%)
	Female	530 (43%)
Smoking status	Smoker	835 (67%)
	Non-smoker	137 (11%)
	Former smoker	274 (22%)
Histological subtypes	Acinar adenocarcinoma	648 (52%)
	Papillary adenocarcinoma	174 (14%)
	Micropapillary adenocarcinoma	75 (6%)
	Solid adenocarcinoma	87 (7%)
	Mucinous invasive adenocarcinoma	50 (4%)
	NSCLC, NOS	125 (10%)
	NSCLC, favoring adenocarcinoma	87 (7%)
pTNM stage		
	I	50 (4%)
	II	87 (7%)
	III	274 (22%)
	IV	835 (67%)

## Data Availability

No new data were created or analyzed in this study. Data sharing is not applicable to this article.
